# CoSMoS: Conserved Sequence Motif Search in the proteome

**DOI:** 10.1186/1471-2105-7-37

**Published:** 2006-01-24

**Authors:** Xiao I Liu, Neeraj Korde, Ursula Jakob, Lars I Leichert

**Affiliations:** 1 Department of Molecular, Cellular & Developmental Biology, University of Michigan, 830 N. University, Ann Arbor, MI 48109-1048, USA

## Abstract

**Background:**

With the ever-increasing number of gene sequences in the public databases, generating and analyzing multiple sequence alignments becomes increasingly time consuming. Nevertheless it is a task performed on a regular basis by researchers in many labs.

**Results:**

We have now created a database called CoSMoS to find the occurrences and at the same time evaluate the significance of sequence motifs and amino acids encoded in the whole genome of the model organism *Escherichia coli *K12. We provide a precomputed set of multiple sequence alignments for each individual *E. coli *protein with all of its homologues in the RefSeq database. The alignments themselves, information about the occurrence of sequence motifs together with information on the conservation of each of the more than 1.3 million amino acids encoded in the *E. coli *genome can be accessed via the web interface of CoSMoS.

**Conclusion:**

CoSMoS is a valuable tool to identify highly conserved sequence motifs, to find regions suitable for mutational studies in functional analyses and to predict important structural features in *E. coli *proteins.

## Background

The number of newly sequenced genes has been growing exponentially over the last decades [1]. This makes it technically impossible to use experimental biology to assign functions and to investigate the regulation of these newly discovered proteins. Over the past years, computational biology has been shown to be a powerful tool to assist in these assignments. This is based on the fact that proteins that share high sequence similarity, either within one organism or between different organisms, often perform very similar functions. Thus, the function of unknown proteins can often be directly predicted using a homology search against a database of proteins with assigned functions. Powerful algorithms and search tools such as BLAST have been developed to perform these homology searches [2,3].

The data derived from these homology searches also provides valuable information about the evolutionary conservation of every single amino acid in the sequence. The neutral theory of molecular evolution states that mutations in amino acids occur in a stochastically constant manner as long as the mutations have no effect on the function of the gene product [4]. On the other hand, amino acids that are important for protein function and structure cannot mutate without a detrimental effect on protein activity. Therefore, these amino acids will change very slowly in a given protein family during evolution. This allows identifying functionally or structurally important residues simply by comparing multiple homologous sequences that stretch a great evolutionary distance and singling out amino acids that are highly conserved or even invariant. Mutational analysis of these amino acids can be used in an experimental setting to unravel the function of the protein.

Patterns of amino acids are, when highly conserved, often important functional or structural features. Although it is relatively easy to find specific patterns of amino acids encoded in the genome of an organism with a simple text or regular expression search, predictions about the conservation of this sequence motif and therefore its biological significance are more difficult [5]. The occurrence of small amino acid sequence motifs encoded in a genome can be purely statistical [6]. But if a sequence motif is highly conserved over a wide stretch of evolutionary distant proteins, it is likely that it plays an important role as part of an active site, a site of regulation or a substrate and cofactor binding site. The CoSMoS motif search tool takes conservation information derived from a comprehensive set of alignments into account and does not only find all occurrences of a small sequence motif in the *E. coli *proteome but it orders these occurrences by evolutionary significance, an important feature that can be used to distinguish between biologically significant and statistical occurrences of a sequence motif.

## Construction and contents

**Figure 1:**
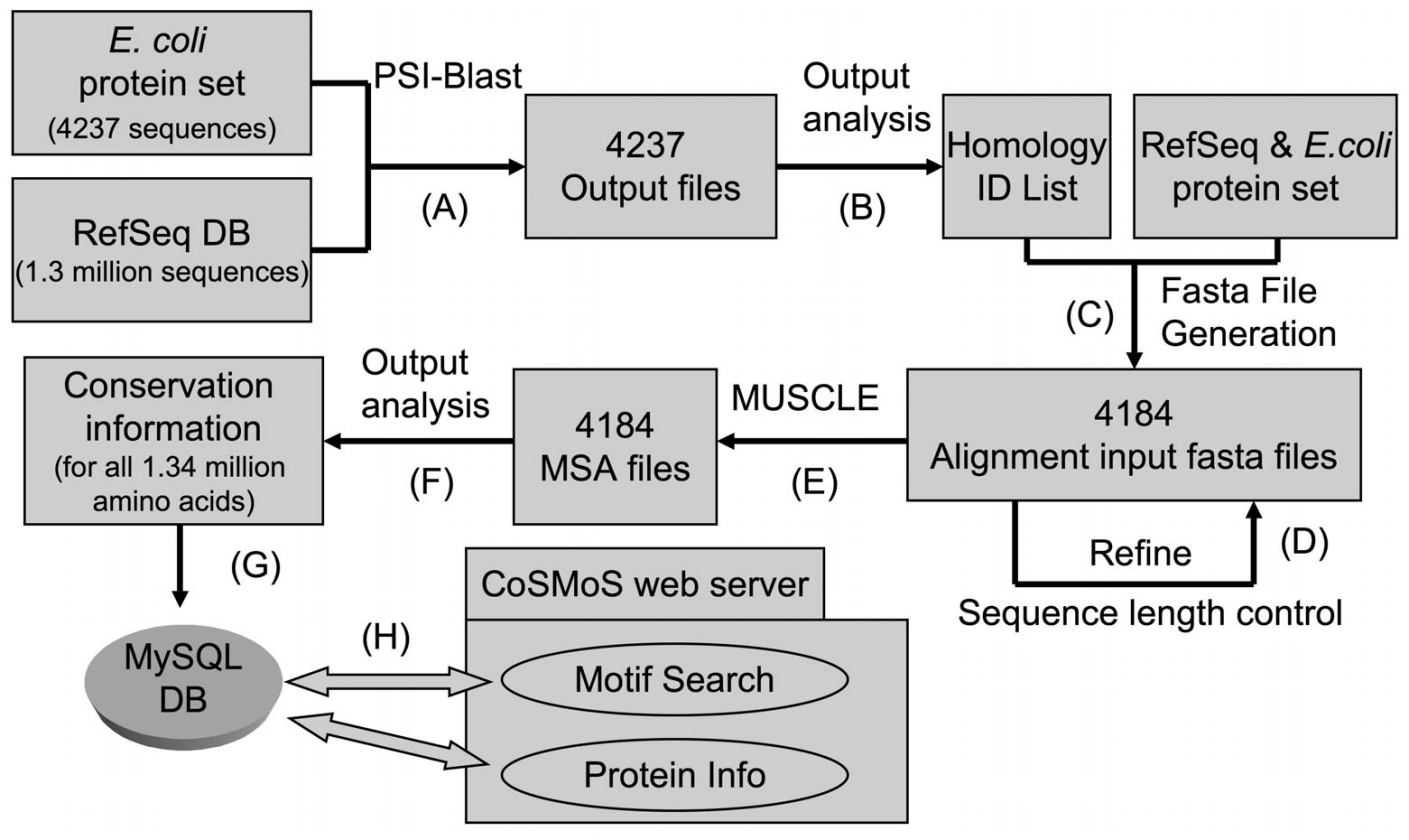
**Construction of CoSMoS**. PSI-BLAST was used to identify homologues of *E. coli *K12 proteins in the RefSeq database (A). The PSI-BLAST output was parsed (B) and used to generate a fasta file for each individual *E. coli *protein containing the *E. coli *sequence itself and all homologous sequences (C). Fasta files were edited (D) to accommodate the MUSCLE alignment (E). Multiple Sequence Alignments (MSA) were then analyzed to extract amino acid (AA) conservation information (F) that was stored along with the according protein information in a MySQL database (G). The MySQL database can be queried using the web frontend [11] (H).

CoSMoS contains homology data for each of the 4237 proteins encoded in the *Escherichia coli *K12 complete genome. By homology analyses based on a comparison to the RefSeq database we could calculate the conservation of each individual of the 1,350,094 amino acids encoded in the *E. coli *genome (Fig 1). Combined with a set of web based accession tools, this database provides a very convenient and fast way to find highly conserved patches, sequence motifs and important amino acids in all the proteins of the model organism *E. coli*. CoSMoS can be used as a tool to find suitable regions for mutation in functional biochemical analyses, to predict important structural features and to identify proteins with highly conserved sequence motifs.

### Homology search

The underlying dataset for the CoSMoS database is the NCBI RefSeq database Release 9 [7]. The *E. coli *K12 complete genome protein sequence (RefSeq reference number NC_000913.2), consisting of 4237 protein sequences, was used as the query for a PSI-BLAST against the complete RefSeq protein database, which contains more than 1.3 million protein sequences from 2780 different organisms. The PSI-BLAST was performed with two iterations and the maximum output of sequences was limited to 2500. The standalone BLAST version 2.2.10 was used in this study. The BLAST output was used to generate a fasta file for each individual *E. coli *protein containing the *E. coli *sequence itself and all RefSeq entries that matched in the PSI BLAST search with an E-value of 10^-5 ^or better. For 498 *E. coli *proteins more than 2499 homologue sequences were found in the PSI-BLAST search that fell below the E-value cutoff.

### Multiple sequence alignment

The fasta files were aligned using MUSCLE, a novel algorithm which creates multiple sequence alignments [8]. MUSCLE was chosen because of its high speed in aligning large sequence sets while still achieving an average accuracy comparable to CLUSTALW [9]. The calculations of the multiple sequence alignments were performed on 6 nodes of the nyx opteron cluster at the Center for Advanced Computing at the University of Michigan. The accommodation of the global alignment calculation by MUSCLE on our hardware required to edit some of the fasta input files. Sequences > 800 amino acids were removed if they were at the same time more than 1.2 fold longer than the corresponding *E. coli *query sequence. This editing resulted in the removal of 1.4% of the total number of sequences in the dataset.

### Database

The multiple sequence alignment for each individual *E. coli *protein was analyzed. For each amino acid of a given protein, the actual number of identical amino acids in the multiple sequence alignment at that position was extracted. These values were stored along with the relevant sequence information in a MySQL database.

## Utility and discussion

The complete CoSMoS database is available as a download [10]. The CoSMoS website can be used to query and search the CoSMoS database in various ways [11]. The output of the search program is highly customizable, allowing the user to refine the search and to modify the output options extensively and, thus, tailor the output to the specific purpose. In addition, default options were set for all tools that provide results in an understandable and easy to follow manner, requiring only little interaction from the user.

### CoSMoS Motif Search

CoSMoS Motif Search is a powerful regular expression based tool that allows users to rapidly identify highly conserved sequence motifs in all proteins encoded in the *E. coli *genome. The output of proteins that harbor this motif is ordered by evolutionary relevance. Using the default settings, the evolutionary significance of a specific set of amino acids is calculated by using both the absolute number of identical or similar amino acids at that given position and the total number of homologous sequences. This is necessary to avoid favoring proteins with only few matches in the homology search (typically in the *Escherichia*, *Salmonella* and *Shigella* genus) but very high sequence similarity. Simply using the absolute number of matching amino acids was also insufficient, because this favors the proteins, which have large numbers of homologues in the RefSeq database. For these reasons we chose an algorithm that ranked the results by both the absolute conservation value and the relative conservation value:

1 a.) Count the number of homologous sequences that have an identical or similar amino acid at the specified position ("absolute conservation score").

1 b.) Divide the "absolute conservation score" by the total number of homologous proteins ("relative conservation score")

2 a.) Rank the motifs by the "absolute conservation score" ("absolute rank").

2 b.) Rank the motifs by the "relative conservation score" ("relative rank").

3.) Assign to each motif the worse (numerically greater) of the 2 ranks ("assigned rank").

4.) Rank the motifs again by their "assigned rank" ("overall rank"). If 2 motifs have the same "assigned rank", the motif with the better (numerically lower) "relative rank" gets the better "overall rank".

**Figure 2:**
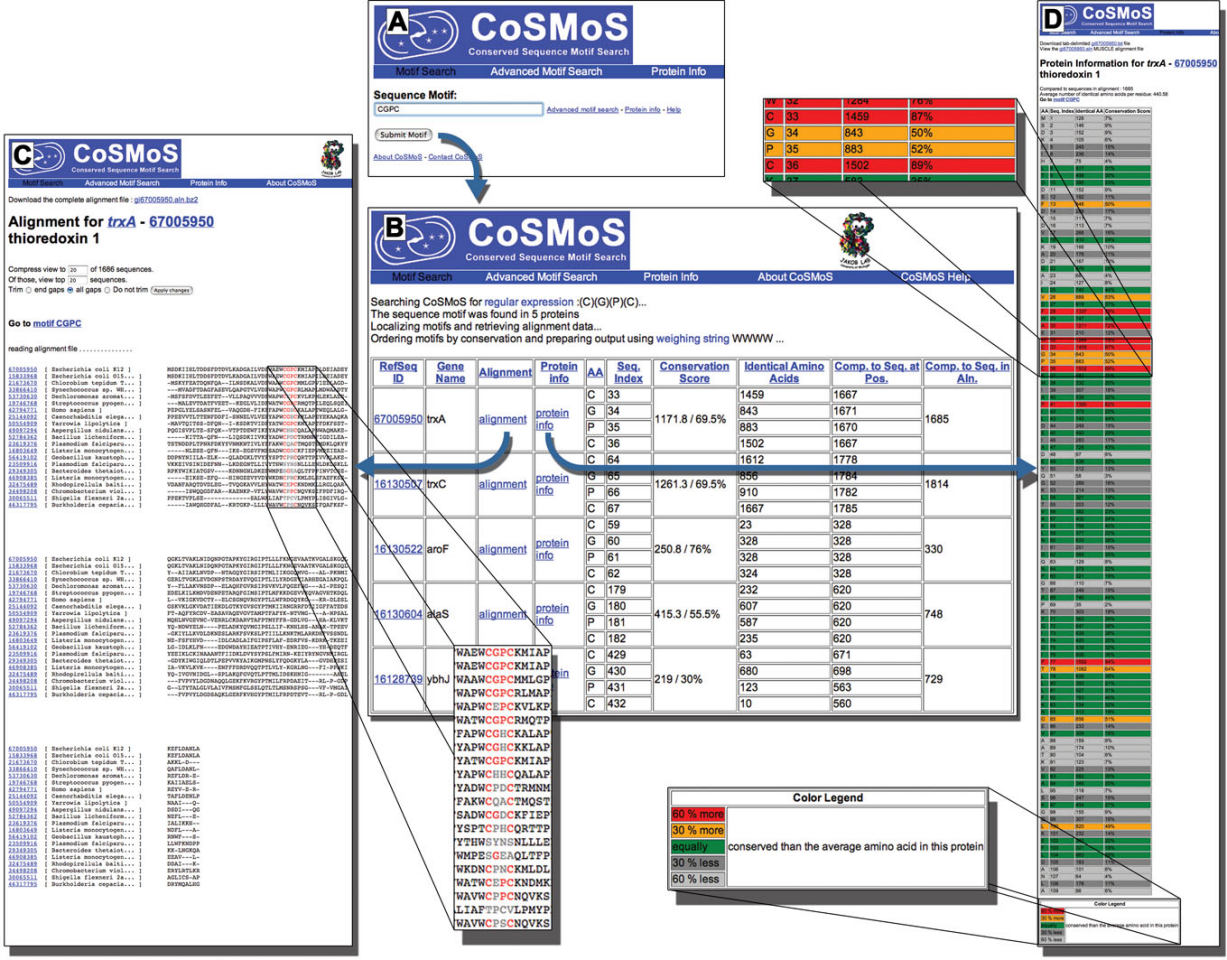
**The CoSMoS website**. (A) The Motif Search web tool can be used to search for small sequence motifs. (B) The result is displayed in a table containing information about the conservation of the amino acids in the motif and links to the alignment (shown here with the "trim all gaps" option) (C) and the CoSMoS gene info page (D).

The output of the CoSMoS motif search consists of a table displaying all occurrences of the motif ordered by the "overall rank" (Fig. 2B). The absolute and relative conservation scores are based on a weighing string that can be modified by the user in the "advanced motif search", for instance to include similar amino acids or to exclude certain parts of the motif from being considered in the calculation.

Other internet based resources for motif identification like *e*MOTIF-SEARCH and ScanProsite are using a different approach [12,13]. These programs are protein centric and require the input of a protein sequence, which is then scanned for known motifs or user defined motifs. It provides the user with the instances these motifs are found in the sequence. Our approach is motif centric and requires the input of a motif that has been described in the literature or was defined using tools like *e*MOTIF-MAKER or MEME [12,14]. Then the whole proteome is searched for that specific motif and all the instances are displayed ordered by their evolutionary significance to make it easy for the user to distinguish between proteins with a purely statistical and proteins with a functionally relevant occurrence of this motif. A somewhat similar tool is eMOTIF-SCAN that does a regular expression search in the SwissProt database, but it lacks the crucial capability to rank these proteins by the significance of the protein sequence [12].

A link to the NCBI RefSeq entry and the CoSMoS info page for the protein in question as well as a link to the alignment that was used to calculate the evolutionary significance is provided. If desired, the often extremely large multiple sequence alignments can be viewed in full, the standard output, however, shows only the multiple sequence alignment compressed to the 20 most diverse sequences (Fig. 2C).

### CoSMoS Protein Info

CoSMoS Protein Info is a tool that shows homology information for individual proteins and is searchable by gene name or RefSeq ID [15]. For each *E. coli *protein, a page is displayed that provides information about the conservation of every amino acid in this protein (Fig. 2D). The output is color coded, ranking from red for amino acids that are most highly conserved through orange, green and finally to grey for amino acids that are highly variable. Thus, highly conserved and presumably important regions of the protein are obvious as brightly colored patches in the sequence (Fig. 2D). The underlying alignment that was used to calculate the homology information for the displayed protein can also be viewed and a link to the NCBI RefSeq entry is provided.

### The thioredoxin motif as example

One possible application of CoSMoS is to search for conserved sequence motifs in *E. coli *proteins. A search for "CGPC" (Fig. 2A), the active site motif for thioredoxins reveals five *E. coli *proteins harboring exactly this motif. Broader definitions of this motif that would also find similar motifs can be implemented using regular expressions that are explained in the help section, however it should be noted that CoSMoS is finding matches solely based on the primary sequence and does not incorporate structural information. The results are listed in a table with the most relevant results listed at the top (Fig. 2B). Each row in the table represents one occurrence of the motif. Relevant information about the gene name, the conservation of the overall motif and the individual amino acids are given. Links point to the corresponding RefSeq entry, the underlying alignment data, and the CoSMoS protein info page for the protein. The protein with the highest conserved CGPC motif is TrxA (Thioredoxin 1). The CoSMoS protein info page for TrxA displays all the amino acids of Thioredoxin 1, together with a color-coded information about the conservation of this amino acid in comparison to the other amino acids in TrxA (Fig 2D). Amino acids that have higher conservation scores than the average amino acid in the protein are orange and red, whereas highly variable amino acids are displayed in shades of grey. A click on the link to the CGPC motif in question focuses the page on the motif and clearly shows that the region around it is one of the highest conserved features of this protein. A quick scroll through the page reveals other conserved features, which are highlighted in red and orange, for example P77, T78. The link to the alignment-file presents TrxA in a Multiple Sequence Alignment in the context of its homologues (Fig. 2C). A click on the link to the CGPC motif scrolls the page to the relevant position in the multiple sequence alignment.

Other proteins found include TrxC (Thioredoxin 2) ranked at position 2 with a nearly identical conservation score as TrxA and three lower ranked proteins that are not members of the thioredoxin family of proteins but nevertheless do contain CGPC motifs. An inspection of the alignment of the lowest ranked protein, YhbJ, by clicking on the provided link reveals that the occurence of this motif in this protein is of statistical nature.

### Future developments

We are currently expanding the database to cover species other than *E. coli *K12 including *Saccharomyces cerevisiae *and *Caenorhabditis elegans*. We are also automating the process of database building and will provide the tools necessary to build CoSMoS-like databases.

## Conclusion

CoSMoS is an open and public database that contains a vast amount of evolutionary data derived from a comparison of all *Escherichia coli *K12 proteins with their homologues found in the RefSeq database. The CoSMoS website provides tools to find significant sequence motifs and amino acids of structural or functional importance in the *E. coli *proteome. In addition CoSMoS serves as a valuable library of alignments of *E. coli *proteins and their homologues.

## Availability and requirements

The CoSMoS web tools CoSMoS motif search and CoSMoS protein info are available at the CoSMoS website [11,15].

The MySQL CoSMoS database is available for download [10].

Both resources are free to any user.

## Authors' contributions

XIL compiled and analyzed the data, assembled the database and implemented large parts of the scripts that run the web site, NK worked on the scripts that run the web site and created the initial database that was used as a template for this work. UJ supervised the project and revised the manuscript. LIL conceived and designed the CoSMoS database and wrote the manuscript.

All authors read the manuscript and gave final approval.

## References

[B1] Koonin EV, Galperin MY (2003). Sequence-Evolution-Function: Computational Approaches in Comparative Genomics.

[B2] Altschul SF, Gish W, Miller W, Myers EW, Lipman DJ (1990). Basic local alignment search tool. J Mol Biol.

[B3] McGinnis S, Madden TL (2004). BLAST: at the core of a powerful and diverse set of sequence analysis tools. Nucleic Acids Res.

[B4] Kimura M (1979). The neutral theory of molecular evolution. Sci Am.

[B5] Hodgman TC (1989). The elucidation of protein function by sequence motif analysis. Comput Appl Biosci.

[B6] Altschul SF, Lipman DJ (1990). Protein database searches for multiple alignments. Proc Natl Acad Sci U S A.

[B7] Pruitt KD, Tatusova T, Maglott DR (2005). NCBI Reference Sequence (RefSeq): a curated non-redundant sequence database of genomes, transcripts and proteins. Nucleic Acids Res.

[B8] Edgar RC (2004). MUSCLE: multiple sequence alignment with high accuracy and high throughput. Nucleic Acids Res.

[B9] Edgar RC (2004). MUSCLE: a multiple sequence alignment method with reduced time and space complexity. BMC Bioinformatics.

[B10] CoSMoS database download. http://www.biology.lsa.umich.edu/cosmos/files/cosmos2.sql.bz2.

[B11] CoSMoS. http://www.biology.lsa.umich.edu/cosmos.

[B12] Huang JY, Brutlag DL (2001). The EMOTIF database. Nucleic Acids Res.

[B13] Gattiker A, Gasteiger E, Bairoch A (2002). ScanProsite: a reference implementation of a PROSITE scanning tool. Appl Bioinformatics.

[B14] Bailey TL, Elkan C (1994). Fitting a mixture model by expectation maximization to discover motifs in biopolymers. Proc Int Conf Intell Syst Mol Biol.

[B15] CoSMoS Protein Info. http://www.biology.lsa.umich.edu/cosmos/protein_info.php.

